# Dielectric, Electric, and Pyroelectric Properties of Ba_1−x_Ca_x_TiO_3_ Ceramics

**DOI:** 10.3390/ma17246040

**Published:** 2024-12-10

**Authors:** Kamil Feliksik, Jolanta Makowska, Joanna A. Bartkowska, Tomasz Pikula, Rafał Panek, Oliwia Starczewska, Małgorzata Adamczyk-Habrajska

**Affiliations:** 1 Institute of Materials Engineering, Faculty of Science and Technology, University of Silesia, 75 Pułku Piechoty 1A, 41-500 Chorzow, Poland; kamil.feliksik@us.edu.pl (K.F.); joanna.bartkowska@us.edu.pl (J.A.B.); oliwia.starczewska@us.edu.pl (O.S.); malgorzata.adamczyk-habrajska@us.edu.pl (M.A.-H.); 2 Department of Electronics and Information Technology, Faculty of Electronics and Computer Science, Lublin University of Technology, 38A Nadbystrzycka Str., 20-618 Lublin, Poland; t.pikula@pollub.pl; 3 Department of Construction Materials Engineering and Geoengineering, Lublin University of Technology, 40 Nadbystrzycka Str., 20-618 Lublin, Poland; r.panek@pollub.pl

**Keywords:** Ba_1−x_Ca_x_TiO_3_ ceramics, dielectric properties, electric properties, pyroelectric properties

## Abstract

In this study, we investigate the dielectric, electric, and pyroelectric properties of Ba_1−x_Ca_x_TiO_3_ (BCT) ceramics with compositions of *x* = 0.2, 0.25, and 0.3. The ceramics were synthesized using the solid-state reaction method. A microstructural analysis was performed using scanning electron microscopy (SEM), revealing that calcium concentration influences grain size and morphology, with BCT20 showing larger, hexagonal grains, while BCT25 and BCT30 exhibited smaller, irregular grains. Phase composition and crystalline structure were characterized via X-ray diffraction (XRD), which confirmed the absence of secondary phases and a predominantly tetragonal P4mm structure for BCT20 and BCT25. However, BCT30 showed an additional orthorhombic (Pbam) phase at 5.9 wt. % alongside the dominant tetragonal phase. Dielectric measurements revealed that increasing the calcium concentration shifts the temperature of dielectric permittivity maximum to lower values, correlating with a shift in the ferroelectric–paraelectric phase transition. Pyroelectric measurements indicated the highest pyroelectric current for BCT25, while BCT30 showed the maximum thermally stimulated depolarization current.

## 1. Introduction

Among perovskites with the general chemical formula A^2+^B^4+^O_3_^2^⁻, titanates are particularly interesting due to their ferroelectric properties [1,2]. The most well-known ferroelectric titanate is barium titanate (BaTiO_3_). Barium titanate-based materials are highly valued for their remarkable dielectric, relaxor, semiconductor, piezoelectric, and pyroelectric properties [3,4,5,6,7]. These compounds are used in a wide range of electronic device applications, including capacitors, sensors, actuators, and tunable devices [8,9,10,11]. It is also important to highlight that barium titanate-based compounds are environmentally friendly, lead-free ceramic materials. This is in contrast to the widely used ferroelectric compositions of the PZT (lead zirconate titanate) type, which, due to the toxic effects of lead, can cause significant environmental and health risks, as well as generate unstable compositions [12,13,14,15,16].

To enhance and stabilize properties, doping with appropriate elements is considered one of the most effective techniques [17,18]. In perovskite structures, the A-site can be occupied by rare earth ions (e.g., Nd^3+^, Pr^3+^, La^3+^) with a +3 oxidation state, divalent alkaline earth ions (e.g., Ba^2+^, Ca^2+^, Sr^2+^), or alkali metal ions (e.g., Ag^+^, K^+^, Na^+^) with a +1 oxidation state. The Bsite, on the other hand, is typically occupied by transition metals (e.g., Mn^3+^, Mn^4+^, Ti^4+^, and Al^3+^) [19]. In BaTiO_3_, various isovalent or aliovalent ions of different sizes can be incorporated into the structure. Ionic radii play a crucial role in determining which ions substitute within the crystal lattice. Doping ions with larger ionic radii tend to replace Ba^2+^ ions, while ions with smaller radii are more likely to substitute for Ti^4+^ in the BaTiO_3_ lattice [20]. Experimental studies have shown that introducing transition metal dopants can significantly affect the structural, ferroelectric, and magnetic properties of BaTiO_3_ ceramics [21,22,23,24,25].

Barium titanate ceramics substituted with ions such as Ca^2+^, Sr^2+^, La^3+^, Zr^4+^, Nb^5+^, and others have been, and continue to be, the focus of extensive research due to their diverse applications and compelling ferroelectric and dielectric properties [26,27,28,29]. Because of the similar ionic radii of Ca^2+^ (1.34 Å) and Ba^2+^ (1.61 Å) in 12-fold coordination, barium titanate, when substituted with Ca^2+^, readily forms a complete solid solution, Ba_1_₋_x_Ca_x_TiO_3_ [30,31]. The inclusion of calcium ions as a BaTiO_3_ modifier helps maintain the phase transition temperature (T_C_) by compensating for atomic polarization rather than distorting the Ti–O bonds [32]. Moreover, this substitution stabilizes the tetragonal structure of barium titanate and prevents the unintended formation of the hexagonal phase [3]. The modification with calcium ions enhances the thermal stability of barium titanate by significantly lowering the temperature required for the transition from the tetragonal to the orthorhombic phase, while also improving the dielectric properties of ceramic capacitors [33,34].

Calcium barium titanate (Ba_1−x_Ca_x_TiO_3_BCT) [35] is a solid solution formed from BaTiO_3_ [36,37] and CaTiO_3_ [38]. CaTiO_3_ is known as a typical depressor in BaTiO_3_ ceramics, significantly reducing dielectric losses and the temperature coefficient of the dielectric constant, with only a slight impact on the Curie temperature [39,40,41,42,43].

Studies have confirmed that the ferroelectric Curie temperature (T_C_) of Ba_1−x_Ca_x_TiO_3_remains almost independent of the calcium ion content for compositions with x ≤ xc, where xc is approximately 22 mol% for ceramics obtained through the conventional solid-state reaction process. This stability in T_C_, as BaTiO_3_ is modified with calcium ions, is in sharp contrast to the effects of doping with other elements, which typically leads to a significant decrease in the Curie temperature in modified barium titanate [44]. Furthermore, in single crystals of this system [45], several intriguing phenomena have been observed, including the significant influence of quantum fluctuations on the ferroelectric phase transition at low temperatures, large electric field-induced deformation, and a strong piezoelectric effect at room temperature. These phenomena are closely related to the interaction of dipole moments arising from the displacements of titanium ions, along with dipole moments resulting from the off-center displacements of smaller calcium ions in the larger spaces left by barium ions [46].

These findings are highly significant, both from a fundamental research perspective and for technological applications of piezoelectrics in temperature-resistant devices [47,48,49,50,51,52,53]. As a result, the family of compounds known as calcium barium titanate presents both a substantial challenge and an opportunity for research and application. It is crucial to gain a deep understanding of the doping process for these ceramic materials, as well as the effects of various modifiers on their properties. Equally important is the development of technology for calcium barium titanate, which allows for the controlled tuning of its properties, comparable to that achieved through doping.

## 2. Materials and Methods

Ba_1−x_Ca_x_TiO_3_ ceramics with compositions of *x* = 0.2 (Ba_0.8_Ca_0.2_)TiO_3_−BCT20, *x* = 0.25−(Ba_0.75_Ca_0.25_)TiO_3_−BCT25 and *x* = 0.3–(Ba_0.7_Ca_0.3_)TiO_3_−BCT30 were obtained using substrates such as barium carbonate BaCO_3_ (Avantor, Radnor Township, PA, USA, 99.99%), calcium carbonate CaCO_3_ (Avantor, Radnor Township, PA, USA, 99.99%), and titanium oxide TiO_2_ (Avantor, Radnor Township, PA, USA, 99.99%). The oxide and carbonate substrate powders, after weighing in stoichiometric amounts, were mixed in a mortar and then in a planetary ball millwet with ethanol (Avantor, Radnor Township, PA, USA, 99.99%) and a zirconium–yttrium grinding media of a diameter *d* = 1 cm in polyamide containers for *t* = 24 h. After mixing, the powders were air-dried. Then, the dry powders were calcined in a muffle furnace for *t* = 3 h at temperature *T* = 1273 K.

During the calcination of the powders, the synthesis of the tested material takes place according to the general reaction Equation (1):

(1 − x)BaCO_3_ + xCaCO_3_ + TiO_2_ → (Ba_1−x_Ca_x_)TiO_3_ + CO_2_↑ (1)

The synthesized powders were mixed in a mortar and then again in a planetary ball mill wet with ethanol and zirconium–yttrium grinding media of diameter *d* = 1 cmin polyamide containers for *t* = 24 h. After mixing, the powders were air-dried. The dry powders were pressed on a semi-automatic hydraulic press at a pressure of *p* = 300 MPa in steel dies into compacts of a diameter of *d* = 10 mm. The compacts were placed in corundum crucibles on a bed of alumina Al_2_O_3_ (Avantor, 99.99%). The compacts were sintered in an electric resistance furnace at *T* = 1673 K for *t* = 3h in an air atmosphere. The temperature rise rate was *v* = 300 deg/h. The cooling rate was the speed of the free cooling of the furnace. After cooling the crucibles, the BCT samples were cleaned off the bed using Aqua sandpaper with gradations of P600, P800, and P1500C.

The relative density of the obtained samples was measured by the Archimedes method in distilled water. It ranges from 5.51 g/cm^3^ for BCT20, through 5.67 g/cm^3^ for BCT25, and up to 5.26 g/cm^3^ for BCT30. A microstructure analysis and qualitative and a quantitative EDS analysis of the produced ceramic samples were performed using a JEOL JSM-7100F TTL LV (Akishima, Japan) scanning electron microscope equipped with an EDS attachment.

The phase composition of the prepared samples was analyzed using X-ray diffraction (XRD) with a Panalytical X’pert PRO MPD diffractometer (Eindhoven, The Netherlands) operating in the standard Θ–2Θ configuration. The diffractometer featured a PW 3050 goniometer, a PIXcel 1D detector, and a copper (Cu) source (CuK α = 1.54178 Å). For the phase and structural analysis, HighScore Plus software (3.0e by PANalytical B.V.), utilizing the PDF-2 DL database (2022 edition) from JCPDS-ICDD, was employed.

Dielectric properties were measured on specially prepared samples using a computerized measurement system, which included the Agilent E4980A LCR meter (Santa Clara, CA, USA). The samples, prepared in the form of discs with a thickness of *d* = 0.6 mm and a surface area of 1 cm^2^, had their parallel surfaces coated with conductive silver paste (P-120, provided by the Polish State Mint) using the burn-in technique. To minimize stresses from earlier mechanical processing and promote the relaxation of the initially immobile defects formed during sintering, the samples were pre-heated at *T* = 400 °C for *t* = 30 min before conducting the measurements.

The enthalpy and characteristic temperatures of the studied ceramic samples were determined based on the performed differential scanning calorimetry measurements using a DSC1 Mettler Toledo (Mettler Toledo GmbH, Greifensee, Switzerland). Before the measurements, the BCT25 ceramic powders were precisely weighed using an electronic analytical balance RADWAG AS 60/220/C/2 (RADWAG, Radom, Poland). The weighted materials were encapsulated in 40 μL aluminum crucibles and sealed hermetically. A hermetized crucible was placed inside the DSC1 measuring chamber. During the measurement, the nitrogen was used as a drying gas, which ensured the non-freezing of the measurement chamber. No further protective gas atmosphere was used. The measurements were taken from room temperature (RT) up to *T* = 300 °C with a 10 °C/min heating rate. For each sample, two cycles were performed. The obtained results were analyzed using DSC, and Mettler Toledo delivered dedicated STARe computer software (Version 11.00a).

Temperature dependencies of pyroelectric and thermally stimulated depolarization (TSD) currents were measured using a computerized system, which included a Keithley 6485 picoammeter as an integral part. Before measurements, the samples were polarized by a DC field applied at a temperature of *T*_p_ = 150 °C for *t* = 20 min and during subsequent cooling to room temperature under this field. Then, the samples were heated at a constant rate of 5 K/min through the FE-PE phase transition to a temperature of 773 K. The current flowing through the sample was measured as a function of temperature and time.

## 3. Results and Discussion

### 3.1. X-Ray Analysis

**Figure 1:**
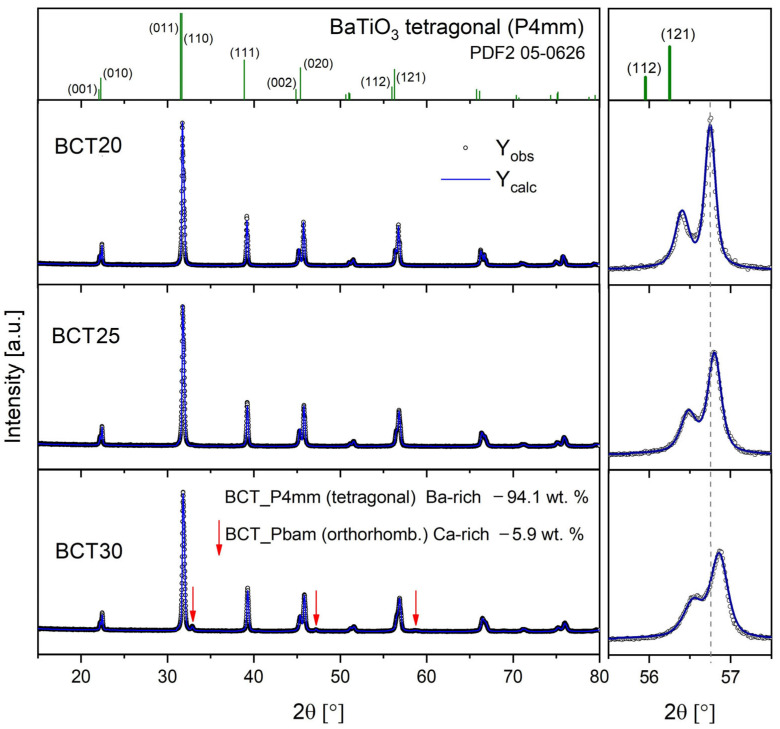
Results of XRD investigations for BCT20, BCT25, and BCT30. The top panel shows a standard pattern for tetragonal BaTiO_3_ for reference (PDF2 card number: 05-0626). The right panel shows the magnification of (112) and (121) twin peaks around the 55.5°–57.5° 2θ angle.

Figure 1 shows XRD patterns registered for the BCT20, BCT25, and BCT30 samples.

It can be noted that pure samples were obtained, as there are no peaks indicating secondary phases. The Rietveld refinement method was used to study the phase composition and crystal structure of the samples. The best numerical fit for BCT20 and BCT25 was obtained, assuming a tetragonal structure and P4mm space group characteristic of α-BaTiO_3_. The standard XRD pattern for the room-temperature form of barium titanate is shown in the top panel of Figure 1 for reference. For BCT30, in addition to the P4mm majority phase, a 5.9 wt. % of the orthorhombic (Pbam) component was detected. The peaks ascribed to the Pbam phase were marked by red arrows in Figure 1. It seems like the solubility limit of Ca in the tetragonal BCT lattice was reached for BCT30 and the additional, Ca-rich, orthorhombic phase was separated. Table 1 summarizes the results of the Rietveld procedure. Significantly reduced lattice parameters for all of the studied samples were obtained, in comparison to the lattice parameters, which reported pure BaTiO_3_ (a = c = 3.9999 A, c = 4.017 A) [54]. This is the effect of the partial substitution of higher Ba^2+^ ions (ionic radius of r_Ba_ = 1.49 A in six-fold coordination) by the smaller Ca^2+^ ones (r_Ca_ = 1.14 A) [55].

It can be noted from the right panel of Figure 1 that the doublets of the (112) and (121) lines shift toward a higher 2θ angle as the concentration of Ca^2+^ increases. In this way, agradual drop in the unit cell volume can be evidenced. The second effect that can be noted from the magnification of the (112) and (121) peaks is their increasing width with the growth of Ca^2+^ concentration. It is caused by microstrain effects introduced by the incorporation of significantly smaller Ca^2+^ ions into the BaTiO_3_ lattice. By applying a size–strain analysis during thefitting of XRD data the following microstrain levels in % were obtained: 0.03, 0.099, 0.144 for BCT20, BCT25, and BCT30, respectively. Moreover, the degree of tetragonality (c/a) also decreases, with the increase in the Ca concentration reaching as low as 1.03%, which is very close to cubic symmetry.

### 3.2. Scanning Electron Microscopy

**Figure 2:**
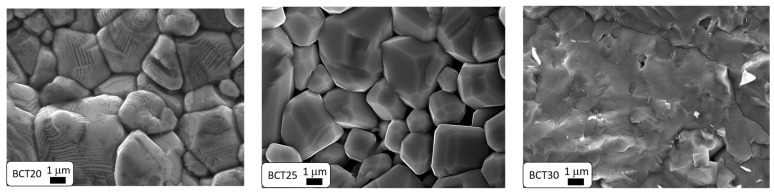
SEM image of Ba_1−x_Ca_x_TiO_3_ ceramics.

Figure 2 presents SEM images of Ba_1−x_Ca_x_TiO_3_ ceramic fractures. The analysis of the ceramic microstructure images reveals significant differences between the tested samples. In the case of BCT20 ceramics, the grains are well-formed, with distinct and relatively well-developed boundaries, showing a tendency toward spiral hexagonal growth [56], which is also characteristic of BaTiO_3_ ceramics [56]. This two-dimensional grain growth mechanism results in a substantial increase in individual grain size, consequently enhancing the strength of the resulting ceramic. The microstructure exhibits a heterogeneous grain distribution, along with a varied grain size. The appearance of the microstructure indicates that, during fracture formation, the cracks propagated through the grains (transgranular fractures) [36]. In contrast, the microstructure of BCT25 ceramics shows grains that are irregular and angular in shape, with clearly defined boundaries. These grains are noticeably smaller compared to those in BCT20 ceramics. For BCT30 ceramics, a higher calcium concentration clearly results in material melting at the grain boundaries. Consequently, the fracture propagates through the grains rather than along the boundaries. 

Table 2 shows the theoretical and experimental percentages of elements (expressed as oxides) for BCT ceramics. The values obtained during the EDS analysis indicate that the chemical composition of the ceramics is consistent with the assumptions. The proportions of individual elements, especially the Ba/Ca/Ti ratio, reflect the intended composition, which confirms that the synthesis process allowed for obtaining a material with the correct stoichiometry. The ceramics maintain the assumed stoichiometry within the accuracy limits of the EDS method, which means that small deviations from the theoretical values are within the tolerances that are characteristic of the method used.

The technological process used during the production of ceramics proved effective in maintaining the stability of the chemical composition. Both in terms of preparing starting powders and the sintering process itself, the technology allowed for controlling the composition of the material and avoiding undesirable impurities or secondary phases.

### 3.3. Dielectric Measurements

**Figure 3:**
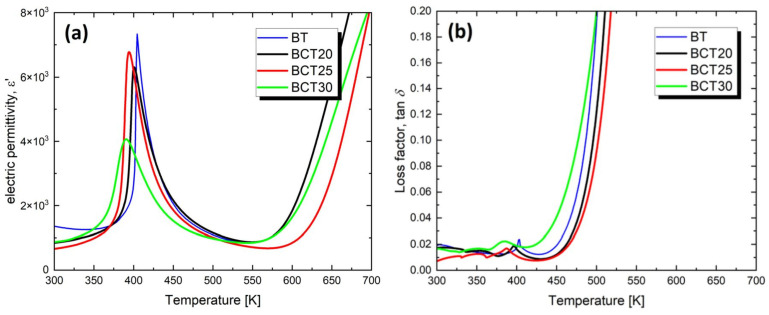
Dielectric permittivity (**a**) and dielectric loss tangent (**b**) as a function of temperature, obtained at a frequency of 1 kHz during the heating process for Ba_1−x_Ca_x_TiO_3_ ceramics.

Such significant differences in the microstructure of the studied compounds were reflected in their dielectric properties. Figure 3 presents the temperature dependencies of the real part of the dielectric permittivity (ε) and the loss tangent (tanδ). 

On the temperature characteristics of electric permittivity (Figure 3a), a distinct maximum is observed, whose temperature decreases with an increasing calcium content (Table 3), which is consistent with the behavior observed by the authors of the study [57,58]. This maximum is slightly higher than the maximum observed in undoped BaTiO_3_ ceramics. The maximum value of electric permittivity for BaTiO_3_ ceramics reported by other authors varies from ε_max_ = 1200 [59] to ε_max_ = 5415 [60], depending on the technological conditions used.

This decrease in Curie temperature is accompanied by a broadening of the ferroelectric–paraelectric (FE-PE) phase transition. Other authors have also noted a decrease in the phase transition temperature with an increasing calcium ion concentration [61]. This behavior suggests that some Ca^2+^ ions substitute for Ti^4+^ ions, contributing to the formation of negatively charged defects Ca″_Ti_ and oxygen vacancies (V^••^_o_) as an electrical compensation for these Ca″_Ti_ defects. The resulting oxygen vacancies locally reduce the tetragonal symmetry, which leads to the aforementioned phase transition broadening and is responsible for the decrease in Curie temperature [62]. The degree of broadening increases significantly in the case of BCT30 ceramics, where the microstructural effect also plays a role, namely, poorly developed, fused grains and the coexistence of tetragonal and orthorhombic structures. These factors lead to the formation of strong stresses in the material, which contribute to the broadening of the phase transition. This negatively affects the ordering of dipoles within domains, which in turn, results in a non-uniform distribution of the polarization direction and, consequently, reduces the value of electric permittivity [63].

**Figure 4:**
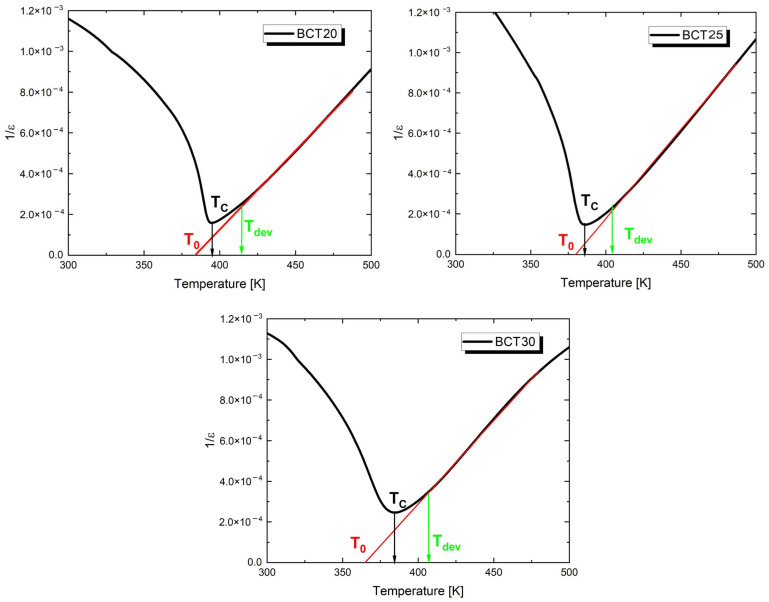
The reciprocal permittivity (1/ε) at 1 kHz as a function of temperature for Ba_1−x_Ca_x_TiO_3_ ceramics.

It is also worth noting the small values of the loss tangent recorded in the temperature range RT-450 K (Figure 3b). These values then begin to increase, which is a consequence of rising electrical conductivity in the samples. It is also worth noting that the lowest losses are exhibited by the BCT25 ceramic (but comparable to undoped BaTiO_3_ ceramic [61]), which is characterized by well-developed but significantly smaller grains compared to BCT20 ceramic. In this ceramic, the share of grain boundaries in the sample volume increases, which confirms the authors’ thesis [63] that grain boundaries have only a minor impact on dielectric losses in the microwave frequency range at low temperatures. So, what causes such low values of the loss tangent observed in BCT25 ceramic compared to BCT20 and BCT30 materials? According to the authors of this study, the explanation lies in achieving an optimal ratio of calcium to barium, which ensures a very stable crystal structure and an appropriate concentration of the aforementioned crystal lattice defects. In the next step of the dielectric measurement results, the temperature function of the reciprocal of electric permittivity was plotted (Figure 4). The shape of the plots indicated that, in the case of all the discussed samples, the variation of electric permittivity has a linear character only from the temperature called T_dev_. Above this temperature, the Curie–Weiss law was successfully applied, which allowed for the determination of the Curie temperature (T_0_) and the Curie–Weiss constant (C). The obtained values of T_dev_, T_0_, and C are collected in Table 3.

The width of the temperature range in which a deviation from the Curie–Weiss law exists does not change significantly with an increase in the calcium concentration. Specifically, for BCT20, the width of this region is approximately 22 K, whereas for BCT25 and BCT30, this value is 19 K and 23 K, respectively. In this region, the modified Curie–Weiss law was applied [64,65,66] (2).

(2) $$\frac{1}{\varepsilon ’}-\frac{1}{{\varepsilon ’}_{max}}=\frac{{\left(T-T_{C}\right)}^{\gamma }}{\delta }$$

where

ε_max_—maximum value of electric permittivity;
δ—the Curie-like constant;
γ—the diffuseness parameter.

**Figure 5:**
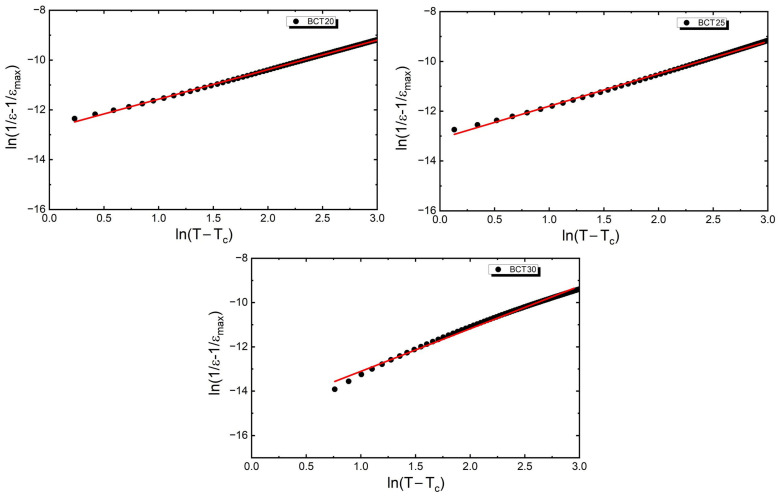
Plot of the dependence of ln(1/ε − 1/ε_max_) as a function of ln(T − T_m_) for Ba_1−x_Ca_x_TiO_3_ceramics.

The plots of ln(1/ε − 1/ε_max_) versus ln(T − T_C_) are shown in Figure 5. The dependencies fit well with the line equation, from which the diffuseness parameter has been extracted. The value of the γ parameter gradually increases with the increasing calcium content. For BCT20, γ = 1.18, and the values achieved for the next two discussed samples were 1.30 and 1.92, respectively. The presented values indicate an increasing diffuseness of the phase transition

### 3.4. Thermal Analysis

**Figure 6:**
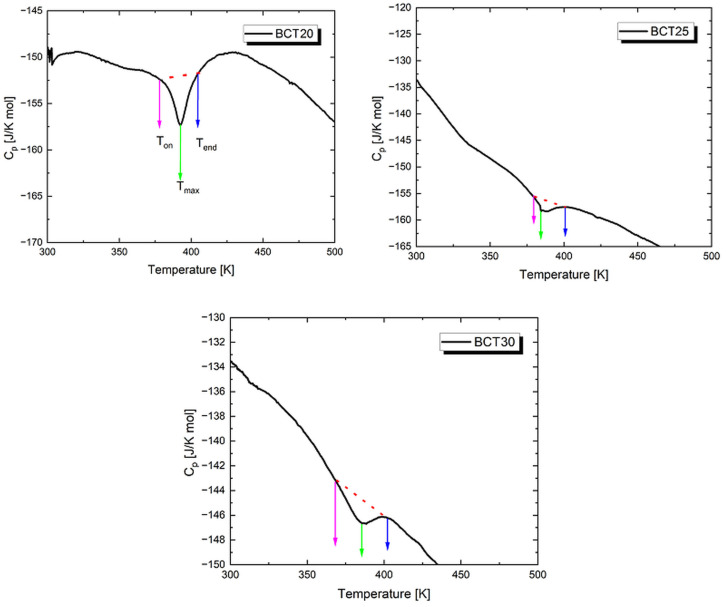
Temperature dependences of the specific heat capacity, Cp, measured upon heating of Ba_1−x_Ca_x_TiO_3_ ceramics with x = 0.2; 0.25; 0.3.

As mentioned above, the maximum observed in the ε(T) characteristics was attributed to the phase transition from the low-temperature ferroelectric phase to the high-temperature paraelectric phase. To confirm this conclusion, the temperature dependence of specific heat was determined through calorimetric measurements. Figure 6 shows the temperature dependencies of the specific heat capacity (C_p_) for all investigated Ba_1−x_Ca_x_TiO_3_ ceramics during heating cycles.

The C_p_(T) curves show an anomaly, which is attributed to the aforementioned ferroelectric–paraelectric phase transition. With an increased calcium ion content, the discussed anomaly becomes more diffuse, reflecting the broadening of the phase transition and remaining in good agreement with the dielectric results presented in the previous section. The temperature range corresponding to this transition, namely the onset temperature (T_on_) and the endset temperature (T_end_), as well as the temperature of the maximum (T_max_), is given in Table 4. The total change in enthalpy was also estimated from the area under the anomaly using the background lines shown in Figure 4 as dashed lines. The results are collected in Table 4. It is worth noting that, in the case of BCT25 ceramics, a significant reduction in enthalpy was recorded, which is likely related to the reduction in grain size. A similar effect was observed for pure BaTiO_3_ and BaTi_0.9_Ge_0.1_O_3_ ceramics [67,68,69,70]. The value of T_max_ is only slightly slower from temperature T_C_ obtained from ε(T) characteristics, which is a commonly known behavior [63,71].

### 3.5. Pyroelectric and Thermally Stimulated Depolarization Currents

**Figure 7:**
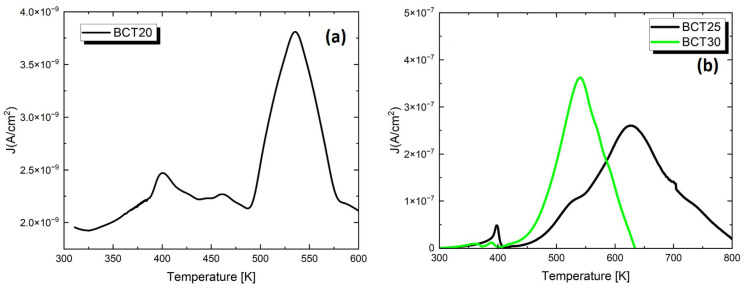
The temperature dependences of pyroelectric and thermally stimulated depolarization currents (TSDC) for Ba_1−x_Ca_x_TiO_3_ ceramics with (**a**) x = 0.2, (**b**) x = 0.25 and 0.3.

To better understand the behavior of dielectric characteristics, as well as to test the application possibilities of the studied materials, measurements of pyroelectric and thermally stimulated depolarization (TSD) currents were conducted. The recorded characteristics are shown in Figure 7.

On each of the presented J(T) characteristics, two maxima are visible. The smaller one occurs at temperatures coinciding with the maximum dielectric permittivity. It can be associated with the peak of the pyroelectric current related to the transition from the ferroelectric phase to the paraelectric phase [72]. It is worth noting that the highest peak value of the pyroelectric current was recorded for BCT25 ceramics. Moreover, on the characteristics I(T) presented in Figure 7, the second distinct maximum is visible, which isconnected tothe presence of thermally stimulated depolarization currents. The existence of thermally stimulated depolarization currents confirms the proposed incorporation of some Ca^2+^ ions in place of Ti^4+^ ions, contributing to the formation of negatively charged defects Ca″_Ti_ and oxygen vacancies (V^••^o) as an electrical compensation for these Ca″_Ti_ defects. Oxygen vacancies are characterized by relatively high mobility because, in the perovskite structure, oxygen ions are always located near the oxygen vacancy, allowing for position exchange. Such highly mobile oxygen vacancies are attracted to oppositely charged centers and oscillate around them, leading to the formation of defect complexes, Ca″_Ti_ − V^••^o. In an unpolarized sample, oxygen vacancies are randomly distributed. Naturally, oxygen vacancies can migrate both within the grains and across the grain boundaries. Considering the model proposed by the authors of study [73], the existence of a TSD current can be explained as follows. When the sample is polarized at a low temperature, vacancies migrate under the influence of the electric field only within the grains. This phenomenon is reflected by a peak observed on the I(T) characteristic in the temperature range of 470 K–540 K for BCT20 ceramics and by a small, suppressed peak in BCT25 ceramics masked by the main peak. This peak originates from the orientation of defect dipoles Ca″_Ti_ − V^••^o, which leads to the relaxation of defect dipoles within the grains that accumulate on the cathode side of the grain boundaries during polarization. In the case of BCT25 ceramics, another main peak is observed on the I(T) characteristic at 640 K. This peak can be attributed to the long-range relaxation resulting from the migration of oxygen vacancies across the grain boundaries, which are depleted in the anodic region and accumulate in the cathodic region after polarization. 

The measurement of temperature-induced changes in pyroelectric currents allows us to determine the pyroelectric coefficient at room temperature. The pyroelectric coefficient at room temperature is highest for BCT25 ceramics, with a value of 1.6 µC/m^2^K, while for BCT20 and BCT30 ceramics, it is 0.32 µC/m^2^K and 0.2 µC/m^2^K, respectively. This is not a high value. However, it should be emphasized that the ceramic polarization process was conducted in a relatively low field of just 0.1 MV/m. In the literature, the values of the pyroelectric coefficient range from 165 µC/m^2^K (for (K_0.5_Na_0.5_)_0_._96_Li_0_._04_O_3_ ceramics [63]) to 414 µC/m^2^K (for PZT ceramics [63]) under polarizing field values of 5 MV/m.

The BCT25 ceramic is characterized by the highest gamma coefficient at room temperature and also exhibits the largest maximum pyroelectric current, making it the most promising base compound for further modifications aimed at sensor applications, specifically pyroelectric sensors. Therefore, the calculations of the figures of merit values (related to its use as pyroelectric sensors) were conducted only for this material [74,75]. Knowledge of these values is essential for assessing the efficiency of converting thermal changes into an electrical signal. It is worth emphasizing that the quantities discussed do not depend solely on the pyroelectric current. In their calculation, the material’s specific heat capacity, the value of electric permittivity, and the dielectric losses are also taken into account. The first one is the current responsivity figure of merit (F_i_), which allows for evaluating the ability of the material to generate an electric current in response to a temperature change. The coefficient is defined by the following relationship (3):

(3) $$F_{i}=\frac{p}{C_{v}}$$

where C_v_ and *p* denote the specific heat capacity of the material and pyroelectric coefficient, respectively [76,77]. In turn, the coefficient that evaluates the ability of a material to generate voltage in response to a temperature change is the voltage responsivity figure of merit (F_v_), which is described by the following formula [78] (4):

(4) $$F_{v}=\frac{p}{C_{v}\varepsilon_{0}\varepsilon }$$

ε_0_ and ε are the permittivity of free space and the electric permittivity of the discussed material, respectively. The last coefficient relating to the material’s sensitivity is the detectivity figure of merit (F_d_). The coefficient is described by the formula [79,80] (5):

(5) $$F_{d}=\frac{p}{C_{v}\sqrt{\varepsilon_{0}\varepsilon tan\delta }}$$

where tanδ is the dielectric loss tangent.

High values of F_d_ are associated with the sensor’s high detectivity, which means it can detect smaller temperature changes.

The values of the mentioned coefficients calculated for BCT25 ceramics are summarized in Table 5. 

The F_d_ value is approximately 10 times lower, whereas F_i_ and F_v_ are 100 times lower, compared to those obtained for the most efficient lead-free ceramic materials, such as [Bi_0.5_ (Na_0.94_K_0.05_Li_0.016_)0.5]_0.95_Ba_0.05_TiO_3_ ceramics [74]. However, it should be noted that the ceramic described in this study was polarized in fields that were 50 times lower. This fact suggests that BCT25 ceramics could be a starting point for further research aimed at increasing the piezoelectric coefficient while simultaneously reducing specific heat, electric permittivity, and the dielectric loss tangent.

## 4. Conclusions

The Ba_1−x_Ca_x_TiO_3_ ceramic powder was synthesized using the mixed oxide method, involving a stoichiometric mixture of oxides and carbonates, including BaCO_3_, CaCO_3_, and TiO_2_. The XRD analysis confirmed the purity of the samples, with no secondary phases detected, and revealed that BCT20 and BCT25 fit best with a tetragonal P4mm structure typical of α-BaTiO_3_, while BCT30 exhibited a 5.9 wt. % orthorhombic (Pbam) phase alongside the dominant P4mm phase. The SEM analysis reveals distinct microstructural differences among the samples, with BCT20 showing well-formed, larger grains with hexagonal growth patterns, enhancing the ceramic’s strength due to transgranular fracture propagation. Conversely, an increased calcium concentration in BCT25 and BCT30 results in smaller, irregular grains and a tendency for material melting. EDS analysis confirms that the produced Ba_1_₋_x_Ca_x_TiO_3_ ceramics retain the intended chemical composition. Dielectric and thermal measurements of Ba_1_₋_x_Ca_x_TiO_3_ ceramics with x = 0.20, x = 0.25, and x = 0.30 revealed a significant impact of calcium content on the properties of the studied materials. Differences in calcium content have a clear influence on the value of dielectric permittivity across the entire temperature range studied. The temperature of the dielectric permittivity maximum, correlated with the phase transition from the ferroelectric to the paraelectric phase, shifts to lower values as the calcium concentration increases. BCT20 and BCT25 ceramics exhibit rather sharp phase transitions, indicating a lack of structural stress and good ferroelectric ordering. In contrast, BCT30 ceramics show diffuse phase transitions caused by microstructural stresses resulting from poorly developed grains and the coexistence of two crystalline phases. The thermal analysis confirmed the ferroelectric–paraelectric phase transition, and the anomalies in specific heat were consistent with the temperatures recorded in dielectric measurements. Measurements of pyroelectric currents and thermally stimulated depolarization currents (TSDC) provided further insights into the material’s behavior, showing that the highest pyroelectric current was recorded for BCT25 ceramics, while BCT30 showed the maximum value of the thermally stimulated depolarization current. Despite relatively low values of the pyroelectric coefficient, the results suggest that modifications such as doping could improve the pyroelectric properties of these materials, increasing their potential for applications in pyroelectric sensors. In summary, BCT ceramics exhibit promising ferroelectric and dielectric properties, especially at a calcium concentration of x = 0.25, but further modifications are necessary to optimize their properties for practical applications.

## Figures and Tables

**Table 1:** Structural data for BCT20, BCT25, and BCT30 derived from Rietveld refinement method. The data for pure BaTiO_3_ were taken from [54] and included in the table for reference.

Sample	Crystal System	Space Group	Lattice Param. [A]	Volume [A^3^]	Tetragonality [%]	Microstrain [%]	Contribution [wt. %]
BaTiO_3_ [54]	Tetragonal	P4mm	a = 3.9999 c = 4.0085	64.13	2.15	---	---
BCT20	Tetragonal	P4mm	a = 3.9638 c = 4.0085	62.98	1.13	0.030	100
BCT25	Tetragonal	P4mm	a = 3.9585 c = 3.9995	62.67	1.04	0.099	100
BCT30	Tetragonal	P4mm	a = 3.9558 c = 3.9966	62.54	1.03	0.144	94.1
	Orthorhombic	Pbam	a = 5.4153 b = 5.4640 c = 7.7074	228.06	---	---	5.9

**Table 2:** Theoretical and experimental summary of substrate content for Ba_1−x_Ca_x_TiO_3_ ceramics, expressed as oxides.

x	Theoretical Substrate Content [%]			Content of Substrates from EDS [%]		
	BaO	TiO_2_	CaO	BaO	TiO_2_	CaO
BCT20	57.38	37.365	5.247	57.74	36.83	5.43
BCT25	55.053	38.235	6.712	55.40	37.57	7.03
BCT30	52.608	39.147	8.246	52.51	39.44	8.05

**Table 3:** The influence of calcium ion content on the dielectric parameters of Ba_1−x_Ca_x_TiO_3_ ceramics determined at a measurement field frequency of 1 kHz.

Ceramic Material	T_C_ [K]	ε_RT_	ε_max_	tgδ_RT_	tgδ_TC_	T_0_ [K]	C[K]	T_dev_ [K]
BCT20	395.8	845	6302	0.017	0.018	371.8	1.3 × 10^5^	417.2
BCT25	387.4	658	6772	0.007	0.017	364.1	1.1 × 10^5^	406.6
BCT30	384.0	867	4064	0.017	0.022	362.4	1.2 × 10^5^	407.2

**Table 4:** The influence of calcium ion content on the parameters of the FE-PE phase transition in Ba_1−x_Ca_x_TiO_3_ceramics.

Ceramic Material	ΔH [J/g]	T_on_ [K]	T_end_ [K]	T_max_ [K]
BCT20	0.52	382.3	402.1	392.4
BCT25	0.26	362.5	404.7	384.3
BCT30	0.40	361.6	401.3	383.5

**Table 5:** Figures of merit of the BCT25 ceramic.

F_i_ [×10^−12^ m/V]	F_v_ [m^2^/C]	F_d_ [μPa^1/2^]
0.9	1.49 × 10^−4^	1.36

## Data Availability

Data are contained within the article. Further inquiries can be directed to the corresponding author.
